# The Early Diagnostic Dilemma in Angioimmunoblastic T Cell Lymphoma with Excessive Plasma Cells Proliferation

**DOI:** 10.1155/2021/9951122

**Published:** 2021-07-16

**Authors:** Chunyan Wang, Xia Mao, Songya Liu, Cheng He, Ying Wang, Li Zhu, Yangyang Wang, Yicheng Zhang

**Affiliations:** ^1^Department of Hematology, Tongji Hospital, Tongji Medical College, Huazhong University of Science and Technology, Wuhan, China; ^2^Clincal Trial and Research Center, Tongji Hospital, Tongji Medical College, Huazhong University of Science and Technology, Wuhan, China; ^3^National Demonstration Center for Environmental and Planning, College of Environment & Planning, Henan University, Kaifeng 475004, China

## Abstract

**Background:**

Angioimmunoblastic T cell lymphoma (AITL) is an aggressive Epstein–Barr virus-associated T cell lymphoma. Clinical syndromes of AITL are not confined to fever and lymphadenopathy, and patients may initially present with polyclonal plasma cell proliferation, which may obscure the underlying disease of AITL, delaying diagnosis. *Case Presentation*. Here, we report two AITL patients with excessive plasma cell proliferation in the bone marrow, peripheral blood, and ascites even mimicking plasma cell leukemia. Both of them had poor endings.

**Conclusions:**

Our report emphasizes the complexity of the clinical manifestations of AITL, which aims to increase the alertness of physicians and improve the rate of early diagnosis. Integrated diagnostic approaches such as histopathology, flow cytometry, cytogenetics, and molecular biology are essential for accurate diagnosis and precise therapy.

## 1. Introduction

Angioimmunoblastic T cell lymphoma (AITL) is an Epstein–Barr virus-associated neoplasm of CD4+ T follicular helper cells (TFH) [1, 2]. Patients usually present with B syndrome, generalized lymphadenopathy, hepatosplenomegaly, and immune-related syndrome/signs. Polyclonal hypergammaglobulinemia and various autoimmune manifestations are distinct features of AITL [3]. Histologically, the architecture of lymph node is partial or complete effacement with proliferation of follicular dendritic cells (FDCs) and high endothelial venules (HEVs). Small to medium-sized neoplastic lymphocytes are present in a polymorphous inflammatory background containing histiocytes, eosinophils, reactive lymphocytes, and immunoblasts. The immunoblasts consist of early stage plasma cell (PC), lymphocytes with plasmacytoid features, and large plasmacytoid cells with blast-like nucleusi [4]. Immunohistochemistry (IHC) showed that the neoplastic T cells express most pan-T cell antigens (e.g., CD3, CD2, and CD5), and at least 2-3 TFH-associated antigens consist of CD4, ICOS, CXCL13, CD279/PD1, CD10, BCL6, and CCR5 [5]. The absence of surface CD3 and the coexpression of CD4/CD10 represent the typical phenotypic aberrancy of AITL by flow cytometry [6–8]. Recurrent mutations in RHOA^G17V^ (Ras homology family member A), TET2 (tet methylcytosine dioxygenase 2), DNMT3A (DNA methyl transferase 3 alpha), and IDH2 (isocitrate dehydrogenase 2, mitochondrial) are genetic features of AITL, followed by mutations of some T cell receptor- (TCR-) related genes [9–11]. IDH2^R172^ mutations are relatively specific to AITL with an incidence of 20–30%, especially co-occurring with chromosome (Chr5) gain [12]. IDH2 mutant cells in AITL may be sensitive to alkylating agents [13]. Cytogenetically, gain of Chr5, Chr3, and Chr21 is often observed in AITL [14, 15].

Reactive polyclonal plasmacytosis occurs in various situations, such as viral infection (EBV, parvovirus B19, and hepatitis), autoimmune diseases, and serum sickness. Excessive plasmacytosis in bone marrow (BM) and peripheral blood (PB) associated with hypergammaglobulinemia in AITL have been reported in the past few years [16–21]. Early diagnosis can be very challenging. Here, we present two male patients with excessive polyclonal PC proliferation in BM, PB, and ascites. Both of them had poor endings. The purpose of this article is to highlight the complexity of the clinical features of AITL to enable physicians recognize the disease at an early stage. Integrated diagnostic approaches such as histopathology, flow cytometry, cytogenetics, and molecular biology are essential for accurate diagnosis and precise therapy.

## 2. Clinical Cases

### 2.1. Case 1

A 69-year-old man with a medical history of atrial fibrillation was admitted to our hospital due to fever for one month. Laboratory tests showed white blood cells (WBC) 14.0 × 10^9^/L, hemoglobin (Hb) 113 g/L, platelet (PLT) count 85.6 × 10^9^/L, and fibrinogen 0.96 g/L. Serum globulin increased to 72.9 g/L. Polyclonal high gamma globulin was observed in serum protein electrophoresis (SPEP) and immunofixation electrophoresis (IFE) (IgG 70.3 g/L, IgA 16.10 g/L, and IgM 3.32 g/L). Chest computerized tomography (CT) revealed generalized lymphadenopathy, significantly enlarged left and right atriums, pulmonary hypertension, and splenomegaly. The 24-hour Holter electrocardiogram monitor showed atrial fibrillation and ST-T changes. Atrial fibrillation was not treated regularly; diltiazem hydrochloride and enoxaparin sodium were suggested by a cardiovascular physician. Concerning the lower fibrinogen and PLT count of the patient, heparin was not given for anticoagulation.

**Figure 1:**
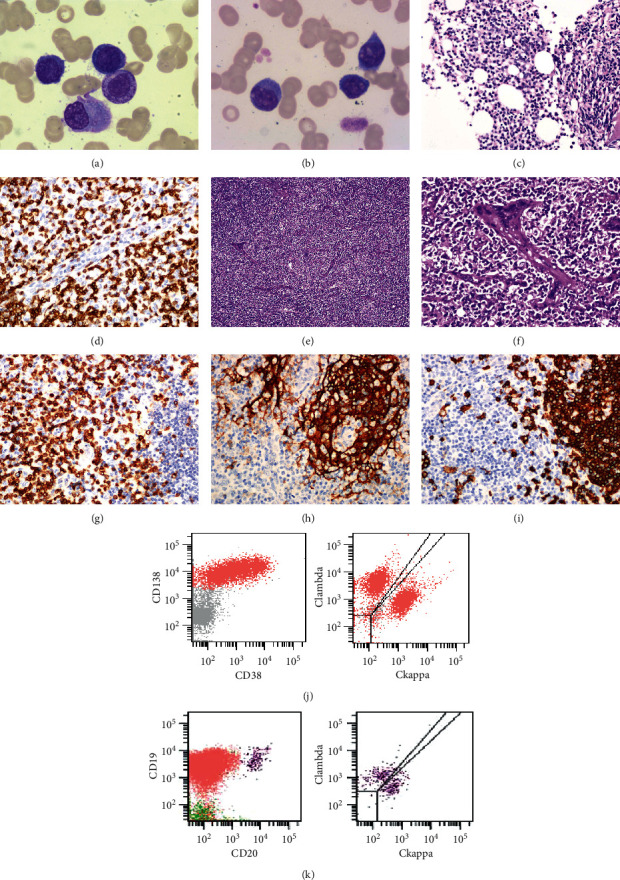
(a) BM aspirates suggesting plasma cell proliferation (Wright's stain) (×1000). (b) Peripheral blood smears showing significantly Rouleaux formation and plasmacytoid (Wright's stain) (×1000). (c) Bone marrow biopsy showing plasma cells proliferation. (d) Plasma cells positive for CD138 (×400). (e) Small to medium-sized atypical lymphoid cells in the lymph node (hematoxylin and eosin stain) (×400). (f) Vascular proliferation (hematoxylin and eosin stain) (×100). (g) Small to medium-sized cells positive for CD3 (×400). (h) CD21-positive follicular dendritic cell meshworks (×400). (i) B cells positive for CD20 (×400). (j) Multiparameter flow cytometry showing polyclonal plasma cells proliferation in the bone marrow. (k) Multiparameter flow cytometry showing polyclonal B cells in the bone marrow.

Morphologically, PC accounted for ∼54% in BM aspiration (Figure 1(a)) and ∼44% in PB smear (Figure 1(b)). Multiparameter flow cytometry (MFC) in BM showed polyclonal PC proliferation with a proportion of ∼45.9% (Figure 1(j)). MFC and TCR/IgH receptor gene rearrangement tests were negative in BM and PB.

After BM and lymph node (LN) biopsies were obtained, the patient received symptomatic treatment and fever was controlled. Plasma exchange could temporarily decrease levels of globulin. One week later, he complained of drowsiness and inability to lift his upper limbs, and thereafter, he gradually developed lethargy and progressive decline in blood oxygen saturation. Cervical LN biopsy suggested angioimmunoblastic T cell lymphoma. Immunohistochemistry (IHC) showed that tumor cells express CD2, CD3, CD5, CD7, CD43, and PD1, and they were negative for CD20, CD10, and BCL6. CD21, CD23, and CD35 showed irregular FDCs proliferation (Figures 1(e)–1(i)). Rare small lymphoid cells were positive for EBV (by EBER in situ hybridization). The results of this patient's examination are shown in Figure 1. The diagnosis of AITL was established, and the patient received low-dose COP chemotherapy (cyclophosphamide 400 mg, vindesine sulfate 2 mg, and dexamethasone 15 mg). However, severe respiratory distress emerged on the third day of chemotherapy, and the patient eventually died of respiratory and circulatory failure.

### 2.2. Case 2

A 53-year-old man was referred to our hospital because of fever and cytopenia for 2 months. He went to a local hospital initially. Laboratory tests showed Hb 83 g/L, PLT count 53 × 10^9^/L, and serum globulin 68.8 g/L. The T-SPOT test was positive. Ultrasound examination revealed right pleural effusion, abdominal effusion, and generalized lymphadenopathy. No acid-fast bacilli or malignant tumor cells were found in ascites. Fine-needle aspiration (FNA) biopsy of left cervical LN was negative. He received a diagnostic antituberculosis therapy, which suspended in the fifth day due to sudden high fever, and pleural effusion was increased. Thereafter, he went to the tuberculosis specialist hospital, and no acid-fast bacilli were found in the pleural and ascites effusion. Finally, two months later, after clinical symptoms appear, he was referred to our hospital. Laboratory tests showed WBC 6.8 × 10^9^/L, Hb 53 g/L, and PLT count 42 × 10^9^/L. Serum globulin was more than 100 g/L. SPEP and IFE demonstrated polyclonal hypergammaglobulinemia (IgA 14.7 g/L, IgG 89.4 g/L, IgM 6.73 g/L, and IgG4 4.96 g/L) and Coomb's test was positive. Human immunodeﬁciency virus, sera cytomegalovirus, hepatitis B serology, and hepatitis C serology were negative. Plasma EBV DNA was 1.19 × 10^3^ copies/ml, and seven days later, it was quantified by qRT-PCR as 3.768 × 10^6^ copies/2.0 × 10^5^ cells, EBV-infected B cells. Symptomatic treatment of anti-infection, red blood cell transfusion, and plasma exchange was failed to prevent disease progression.

**Figure 2:**
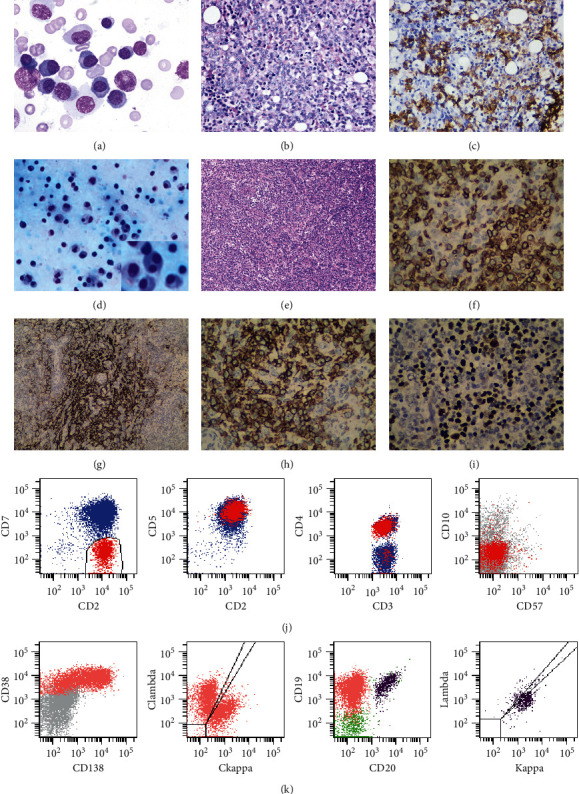
(a) Bone marrow aspirate suggesting Rouleaux formation and plasma cell proliferation (Wrights stain) (×1000). (b) Bone marrow biopsy showing plasma cells proliferation and plasma cells distributed in patches (May–Giemsa stain) (×400). (c) Plasma cells positive for CD138 (×400). (d) Ascites abscess cytology showing large amount of plasma cells with few lymphocytes (Wrights stain) (×400). (e) Small to medium-sized atypical lymphoid cells in the lymph node (hematoxylin and eosin stain) (×400). (f) Neoplasm cells positive for CD3 (×400). (g) CD21-positive follicular dendritic cell meshworks (×400). (h) Neoplasm cells positive for PD-1 (×400). (i) Part of lymphocytes (40–50%) was EBV positive (×400). (j) Multiparameter flow cytometry showing abnormal phenotype T lymphocytes in the lymph node. (k) Multiparameter flow cytometry showing polyclonal plasma cells proliferation in ascites. (l) Multiparameter flow cytometry showing mature B cells with light chain negative in ascites.

LN immunohistochemistry revealed tumor cells with CD3, CD5, PD1, and BCL2 positive and CD10 negative, suggesting AITL, which was verified by ancillary examinations results. The results of this patient's examination are shown in Table 1 and Figure 2. A diagnosis of AITL was established, and the patient received a cycle of R-CHOP regimen (rituximab, cyclophosphamide, doxorubicin, vincristine, and prednisone). Serum globulin was decreased to 84.8 g/L on the fourth day of chemotherapy. However, polyserous effusions and serum globulin levels had not been improved. The blood coagulation disorder is worse than before, and the performance of disseminated intravascular coagulation (DIC) appears. Ultimately, the patient abandoned treatment and discharged.

## 3. Discussion

Clinical procedures of AITL are diverse and aggressive. BM, spleen, skin, and liver are the most common extranodal involvement sites. Effusion/edema/ascites occurs in 25–53% of patients and are usually nonneoplastic. BM involvement can be seen in 28–60% patients [22]. MFC seems to be more sensitive to detect involvement than morphology in BM. The neoplastic T cells ranged 0.1–20% of total cells examined by MFC (median, 0.6%) in morphology-negative patients [6]. The reason may be that AITL often have a rich background of reactive cells, resulting in a low proportion of tumor cells. However, BM aspirate only counts 200 nucleated cells, which may cause a decrease in sensitivity. It is worth noting that flow cytometry still has diagnostic pitfalls in the diagnosis of T-NHL. On the one hand, due to the polymorphism of the phenotype of T lymphocytes, reactive T lymphocytes may have changes in the expression intensity of pan-T antigens. But the clonality of T cells cannot be identified like B lymphocytes using restricted expression of light chains. The detection of T cell receptor *β*-chain constant region 1 (TRBC 1) may be helpful, and its polyclonal expression can play a role in exclusion, but the monoclonal expression of TRBC1 does not confirm that it must be an abnormal clone. On the other hand, in the following situations, the tissue samples are contaminated by formaldehyde or apoptosis too fast, and the cells lose their viability; there are not enough channels to detect pan-T markers, such as four-color flow cytometry; there are not enough cells for analysis, such as fine-needle aspiration samples, or the proportion of abnormal cells is extremely low; or the abnormal T cells only had changes in expression intensity instead of loss of pan-T antigens expression; or the analysts does not have enough experience; all of these circumstances can lead to negative results. As to AITL, according to our preliminary experiences, apart from the above reasons, abnormal T lymphocytes do not express T cell receptor *αβ* in nearly ∼57% (51/90) cases. It is impossible to use TRBC1 to judge the monoclonality of suspicious T cells. In addition, AITL has a subtype of sCD3+CD4+CD7-phenotype, the pan-T markers only loss CD7, the expression intensity of CD3 is isointensity, or slightly diminished compared with normal T cells. There are a small amount of sCD3+CD4+CD7-cells in the normal bone marrow, and the proportion of this group of cells may increase under reactive conditions; this kind of AITL is prone to be missed. The addition of more markers of follicular helper T lymphocytes can be helpful to increase positive rate. At last, the bone marrow invasion of AITL is not 100%, so the negative detection in the bone marrow cannot rule out the diagnosis. In patients without BM involvement, trilineage cytopenias and polyclonal plasmacytosis can be observed as secondary features. In our report, patients presented with hypergammaglobulinemia, anemia, and thrombocytopenia, and one patient had polyserous cavity effusions. BM and ascites examinations were performed prior to the diagnosis of AITL. BM aspirate revealed significantly Rouleaux formation with excessive plasmacytosis, mimicking plasma cell leukemia. Paradoxically, PC and immune globulin were both polyclonal. There was no evidence of BM involvement in both of them. Large B cells without surface and cytoplasm light chain expressed were observed in ascites of the second patients by MFC, resembling diffuse large B cell lymphoma. The negative of IgH receptor gene arrangement was nonsupport. With the experience obtained from the first patient, we suggested the probability of AITL of the second one, and rebiopsy was recommended.

LN biopsy is indispensable but not always available. Integrated diagnostic approaches including MFC and molecular genetics examinations are essential for accurate diagnosis. As to the second patient, histopathology suggested AITL by reexamination of LN, and neoplastic T cells detected by MFC and the clonal of TCR gene rearrangement verified the diagnosis. But the immunophenotyping of tumor cells by MFC in LN were CD2+, CD7−, sCD3+, CD4+, and CD10−, not the typical sCD3−/CD4+ or CD10+ immunophenotyping. It probably cannot distinguish AITL from other peripheral T cell lymphoma. But the usage of MFC can quickly exclude reactive lymphoid hyperplasia, T cell rich large B cell lymphoma, and plasma cell diseases, which are difficult to differentiate by pathology. The NGS test detected the recurrent mutations of TET2 and IDH2^R172k^, which further verified the diagnosis, and may be potential therapeutic targets.

AITL have a dismal prognosis with a five-year overall survival (OS) of 32–41% and a seven-year OS of 30% [23, 24]. Older age (>60 years old), elevated WBC and IgA levels, thrombocytopenia, high international prognostic index and prognostic index for PTCL-U scores, and number of extranodal involvement sites >1 were adverse factors for OS [3, 25, 26]. Patients with AITL with polyserous effusions seem to have poor endings [27, 28]. Plasma cell hyperplasia seems to have no prognostic significance in AITL patients [17]. We reviewed the clinical history, diagnosis, and treatment process to speculate the possible causes of poor endings. The first patient had a history of atrial fibrillation without regular treatment. Excessively elevated PC may lead to blood stickiness, which may increase the burden on the heart and further induce atrial fibrillation. The treatment to prevent thrombosis formation was unavailable because of lower fibrinogen and PLT count. The manifestations of unable to lift limbs, somnolence, and the sudden decline in blood oxygen saturation lead to the hypothesis of cerebral thrombosis and/or pulmonary embolism. Cerebral infarction may be the direct cause of death. We speculate that the underlying cardiocerebrovascular diseases may make prognosis worse in patients with AITL and excessive plasma proliferation, consistent with Kelsey Sokol's report [29]. The second patient presented with severe anemia, thrombocytopenia, and polyserous cavity effusion at onset, excessive polyclonal PC emerged in BM, PB, and ascites without neoplasm T cells infiltration. The T-SPOT test was positive; he was initially misdiagnosed as tuberculosis. The delayed diagnosis time superadded multiple adverse prognosis indicators that lead to poor conditions of the patient.

The relationship between excessive proliferation of PC and AITL has not been expounded comprehensively. TFH is helpful for differentiation of B cells to PC or memory B cells in lymphoid tissues [29]. In the AITL-NGO mouse model, AITL tumor cells were proved to act as TFH [30]. PC proliferation was accompanied by extensive secretion of IL-6, and the secretion of immune globulin can be completely stopped by adding anti-IL-6 and anti-CD126 (IL-6 receptor) monoclonal antibodies, which manifested that IL-6 is necessary for PC proliferation [31]. The ultimate differentiation of EBV-infected B cells into PC was related to EBV replication, which is suppressed by a strong T cell immune response in EBV-seropositive healthy people [32]. EBV-positive B cells can be detected in 66–86% patients of AITL, on account of immunosuppression due to AITL [33]. Tumor T cells, cytokines, and EBV infection may potentially cause polyclonal plasma cell expansion. In our second patient, EBV copies in peripheral blood mononuclear cells was increased to millions, IL-6 levels elevated in sera, and these results were consistent with the past literatures. Whether PC component be a reflection of earlier disease or not should be further explored.

In conclusion, the clinical manifestations of AITL patients are diverse. AITL should be highly suspected in patients presenting with exuberant polyclonal PCs in extranodal sites, combined with syndromes of generalized lymphadenopathy, high EBV copies, and presence of autoimmune antibodies. Integrated diagnostic approaches are essential for accurate diagnosis and precise therapy. Cardiocerebrovascular diseases may be an adverse factor for patients with AITL and significant plasmacytosis, which need to be verified in the future by more samples. The efficacy of traditional treatment in patients with AITL and excessive PC proliferation is less-known and deserves more attention.

## Figures and Tables

**Table 1:** Ancillary examination results of BM, ascites, and LN.

	BM	Ascites	LN
Morphology	PCs were account for ∼48.5%, showing large size, round, or irregular nucleus	Large amount of PCs with immature appearance, some of PC are double nuclei; few lymphocytes scattered	Proliferation of FDCs and HEVs with lots of PCs infiltration and part of lymphocytes (40–50%) were EBV positive

PC (by MFC)	Polyclonal (∼34.7%)	Polyclonal (∼36.3%)	Polyclonal (∼18.3%)

B lymphocytes (by MFC)	Cytoplasm light chain negative (∼0.24%)	Large B cells with surface and cytoplasm light chain negative (∼4.9%)	Normal polyclonal B cells (∼13.6%)

Abnormal T cells (by MFC)	Negative	Negative	Abnormal T cells account for ∼15.3% with CD2+, CD7−, sCD3+, CD4+, and CD10-

Karyotype	Negative	Not done	46, XY [19]/48, XY; +3, +10/44, XY, +3, −9, −10, −15 [1]

Receptor gene rearrangement	Negative	Negative	TCR gene rearrangement positive and IgH rearrangement negative

NGS	Not done	Not done	TET2 gene C1193 W mutation, G1275 R mutation, and IDH2 gene R172k mutation.

BM, bone marrow; LN, lymph node; PC, plasma cell; MFC, multiparameter flow cytometry; FDCs, follicular dendritic cells; HEVs, high endothelial venules; EBV, Epstein–Barr virus; NGS, next-generation sequencing; TCR, T cell receptor; IgH, immunoglobulin heavy chain; TET2, tet methylcytosine dioxygenase 2; IDH2, isocitrate dehydrogenase 2.

## Data Availability

The data used to support the findings of this study are available in the archives of the Tongji Hospital affiliated to Tongji Medical College of Huazhong University of Science and Technology, Wuhan, China.

## Abbreviations

AITL: Angioimmunoblastic T cell lymphoma
TFH: T follicular helper
BM: Bone marrow
PB: Peripheral blood
MFC: Multiparameter flow cytometry
EBV: Epstein–Barr virus
WBC: White blood cells
Hb: Hemoglobin
PLT: Platelet
CT: Chest computerized tomography
ECG: Electrocardiogram
FNA: Fine-needle aspiration
LN: Lymph node.
